# Bacterial social interactions in synthetic *Bacillus* consortia enhance plant growth

**DOI:** 10.1002/imt2.70053

**Published:** 2025-06-08

**Authors:** Yan Liu, Baolei Jia, Yi Ren, Weibing Xun, Polonca Stefanic, Tianjie Yang, Youzhi Miao, Nan Zhang, Yanlai Yao, Ruifu Zhang, Zhihui Xu, Qirong Shen, Ines Mandic‐Mulec

**Affiliations:** ^1^ Jiangsu Provincial Key Lab of Solid Organic Waste Utilisation, Jiangsu Collaborative Innovation Centre of Solid Organic Wastes, Educational Ministry Engineering Centre of Resource‐Saving Fertilisers, Jiangsu Provincial Key Laboratory of Coastal Saline Soil Resources Utilisation and Ecological Conservation Nanjing Agricultural University Nanjing China; ^2^ Xianghu Laboratory Hangzhou China; ^3^ Department of Microbiology, Biotechnical Faculty University of Ljubljana Ljubljana Slovenia; ^4^ Present address: Jiangsu Engineering Research Centre for Soil Utilisation & Sustainable Agriculture, Jiangsu Centre for Collaborative Innovation in Geographical Information Resource Development and Application, School of Geography Nanjing Normal University Nanjing China

**Keywords:** *Bacillus velezensis* SQR9, colonization, *gyrA*, PGPR, social interaction, swarm encounter assay, synthetic *Bacillus* consortia

## Abstract

Plant growth‐promoting rhizobacteria (PGPR) represent a sustainable method to improve crop productivity. Synthetic microbial consortia have emerged as a powerful tool for engineering rhizosphere microbiomes. However, designing functionally stable consortia remains challenging due to an insufficient understanding of bacterial social interactions. In this study, we investigated the effects of *Bacillus velezensis* SQR9 (i.e., a commercially important PGPR) on social interactions within the rhizosphere community, particularly among *Bacillus* species. SQR9 inoculation significantly enhanced cucumber plant growth and altered the structure of rhizosphere *Bacillus* and its related bacterial communities. The results of swarm boundary and carbon utilization assays, revealed that phylogenetically closer *Bacillus* strains exhibited increased social cooperation and increased metabolic niche overlap. Building on these social interactions, we designed 30 consortia comprising both highly related (HR) and moderately related (MR) types across four richness levels (1, 2, 3, and 4 strains), with MR consortia demonstrating superior PGP effects through enhanced plant growth, root colonization, indole‐3‐acetic acid production, and siderophore production, than the HR consortia. Expanding these findings to 300 consortia across four richness levels (1, 2, 4, and 8 strains) confirmed enhanced PGP effects in MR consortia with increasing richness. These findings highlight the importance of bacterial interactions and phylogenetic relationships in shaping rhizosphere communities and designing synthetic microbial consortia. Specifically, this study provides a framework for assembling *Bacillus* consortia that enhance cooperation, which would aid in improving their stability and effectiveness in agricultural applications.

## Highlights

- *B. velezensis* SQR9 inoculation significantly enhanced cucumber plant growth and altered the structure of rhizosphere *Bacillus* and its related bacterial communities, promoting cooperation among closely related strains.
- Phylogenetically closer *Bacillus* strains exhibited increased social cooperation and increased metabolic niche overlap, enhancing community interactions.
- Consortia with moderately related strains exhibited superior plant growth‐promoting effects, including increased plant height, dry weight, root colonization, and production of IAA and siderophores.
- The study provides a framework for assembling *Bacillus* consortia with enhanced cooperation and improving the stability and effectiveness of microbial inoculants in agricultural applications.

## Graphical abstract

Inoculation with plant growth‐promoting rhizobacteria SQR9 enhances cooperative behaviors and compatibility within the microbial community, promoting plant growth. Building on these interactions, synthetic *Bacillus* consortia were designed based on phylogenetic relationships and social cooperation, where moderately related consortia exhibited stronger plant growth‐promoting effects. This design strategy underscores the importance of microbial social dynamics in optimizing synthetic communities for sustainable agriculture.

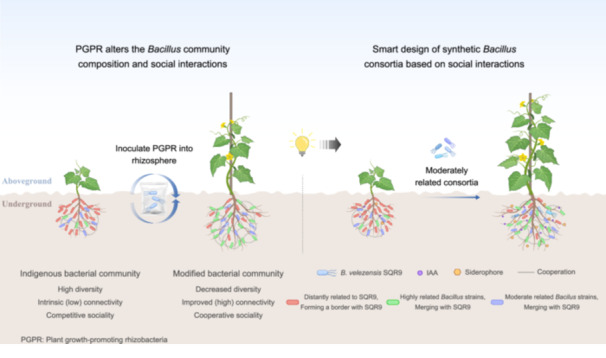

## INTRODUCTION

The rhizosphere microbiome, also referred to as the second genome of the plant, plays a crucial role in plants' growth, nutrition, and overall health of plants [1, 2]. In agriculture, plant microbiota is considered the cornerstone of the next green revolution, as it can improve crop performance while reducing toxic chemical application [3]. Several plant growth‐promoting rhizobacteria (PGPR) strains (e.g., *Bacillus* and *Pseudomonas* spp.) have been used as microbial inoculants owing to their ability to promote plant growth via direct interactions with plants or indirectly by influencing soil microbiota [4, 5, 6, 7]. Microbial inoculants can influence the plant microbiome through various mechanisms, such as altering microbial diversity, promoting microbiome balance, reverting the microbial imbalances caused by pathogens, inhibiting potential pathogens, and boosting the growth of beneficial microbes. Shifting focus to beneficial microbes shows considerable potential; however, their effectiveness is often shaped by the complex social interactions within microbial communities [8, 9]. Compared to single strains, synthetic microbial consortia offer broader functional capacities and improved stability and robustness compared to single strains [10, 11, 12]. However, their consistent plant growth‐promoting effects may be affected by the interactions among constituent strains [13, 14, 15]. Understanding how microbes interact in the rhizosphere can help develop improved strategies to optimize these interactions in synthetic microbial consortia, which are powerful tools for engineering rhizosphere microbiomes.

Microbial communities or individuals within these communities interact with each other and the surrounding cells in multiple ways, resulting in competition, cooperation, or other interactions among neighbors [16, 17]. Within a community, there are numerous negative and positive ecological interactions among individual microbes. Negative interactions may arise from competition for resources and biochemical antagonism [18, 19, 20], and positive interactions may result from the sharing of metabolic products by intra‐ and interspecific community members [21]. Application of microbial inoculants is thought to negatively impact microbial species and strains with overlapping metabolic niches [22, 23]. However, ecological interactions, including competition and antagonism, can also alter the niche space of a particular strain or species, potentially reducing it [24, 25]. Positive interactions, in turn, may lead to the expansion of niche space [26]. However, little is known about the impact of beneficial bacteria in the rhizosphere on social interaction dynamics within the community [27]. In natural rhizosphere environments, indigenous *Bacillus* communities exhibit high genetic diversity and complex population structures [28, 29, 30, 31]. Their members engage in dynamic social interactions (e.g., competition, cooperation, and antagonism [32]) that are regulated by multiple factors, including environmental conditions, nutrient availability, and phylogenetic relatedness [33, 34, 35]. These interactions are highly variable across time and space, making it a major challenge to accurately identify and dissect their effects on community function and plant growth without disrupting the native microbial architecture.

*Bacillus* spp., well‐known spore formers and soil colonizers [15], exhibit complex social behaviors, such as swarming. Swarming is a cooperative movement on surfaces, which plays a key role in root colonization [36]. The *Bacillus subtilis* soil isolates preferentially merge their swarms with close relatives [37, 38]. Such selective behavior involving differential treatment towards relatives by individual microbes is known as kin discrimination (KD) [37]. Within *B. subtilis*, kin soil isolates, defined as strains with at least 99.5% sequence identity at the housekeeping gene level, tend to merge their swarms and invade new territory cooperatively [39, 40, 41]. In co‐incubation experiments, kin cells form mixed biofilms on plant roots, whereas non‐kin cells exclude each other, with one strain primarily colonizing the root surface [40]. However, it remains challenging to relate the phylogenetic shifts in the indigenous rhizo‒*Bacillus* community to social interactions and community function [42].

**FIGURE 1:**
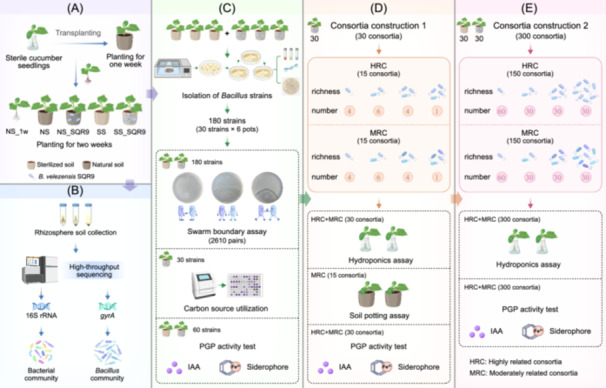
Experimental design and workflow. (A) Cucumber seedlings were transferred from a hydroponic culture system into fresh soil for 1 week (NS_1w). The remaining seedlings were transferred to natural (NS) or sterilized soils (SS), with half of these soils inoculated with *B. velezensis* SQR9 (SS_SQR9 and NS_SQR9). These were incubated for two additional weeks before sampling. (B) Rhizosphere soils from five treatments (NS_1w, NS, SS, SS_SQR9, and NS_SQR9) were collected and DNA was extracted for high‐throughput sequencing of the 16S rRNA gene and *gyrA* genes to characterize both the bacterial and *Bacillus* communities. (C) One‐hundred and eighty *Bacillus* strains were isolated from the rhizosphere soils of the SS and SS_SQR9 treatments. Social interaction assays, including 2610 pairs, were conducted in three batches to evaluate *Bacillus* interactions in different treatments. Carbon source utilization abilities of 30 strains from SS_SQR9 were assessed, aiming to understand the relationship between strain phylogeny and nutrient utilization. Plant growth‐promoting (PGP) activities of 60 strains from SS and SS_SQR9 treatments were analyzed, including IAA and siderophore production. (D) Two types of consortia, HR and MR, were designed, each with four richness levels (1, 2, 3, and 4 strains), resulting in a total of 30 combinations. Greenhouse experiments, including both hydroponic and soil culture setups, as well as PGP activity assays, were conducted to investigate how strain relatedness influences the PGP potential of the microbial communities. (E) 300 consortia combinations were created for extended validation, consisting of HR and MR consortia, each with four richness levels (1, 2, 4, and 8 strains). *gyrA*, DNA gyrase subunit A gene; IAA, indole‐3‐acetic acid; PGP, Plant growth‐promoting.

In this study, we aimed to fill the knowledge gap regarding the relationship between bacterial social behaviors and their PGP activity by examining how the gram‐positive PGPR strain *B. velezensis* SQR9 (hereafter referred to as SQR9) alters social interactions and community function. This well‐studied PGPR strain [43, 44] is known for its outstanding ability to suppress soil‐borne diseases and promote plant growth [23, 38, 39, 40, 41, 42, 45]. We conducted a greenhouse experiment with cucumber plants to investigate whether SQR9 influences the rhizosphere bacterial community structure (Figure 1). Two‐week‐old cucumber seedlings were transferred to pots with natural or sterilized soil and inoculated with SQR9. Plant shoot height, dry weight, and the rhizosphere bacterial community (analyzed using 16S rRNA gene and DNA gyrase subunit A gene (*gyrA*) sequencing) were assessed. *Bacillus* strains were isolated from the rhizosphere, and a swarming boundary assay was performed to evaluate social interactions. Based on phylogenetic relatedness, social behavior, metabolic niches, and PGPR traits, we designed two types of *Bacillus* consortia—highly related (HR) and moderately related (MR). PGP functions of the consortia were confirmed through hydroponic systems, soil pot experiments, and measurements of indole‐3‐acetic acid (IAA) and siderophore production. To further validate this design strategy, we expanded the experiment to include 300 consortia with 4 richness levels (1, 2, 4, and 8 strains). Results confirmed that the MR swarm‐merging strains significantly enhanced PGP activity in a richness‐dependent manner, emphasizing the importance of ecological compatibility and niche breadth in consortium design. These new findings, combined with the existing knowledge of bacterial community structure, phylogenetic relationships, sociality, and PGPR traits of *Bacillus* rhizosphere isolates, provide insights for designing beneficial *Bacillus* communities for plant growth.

## RESULTS

### *B. velezensis* SQR9 alters the bacterial community in cucumber rhizosphere

To investigate the effect of SQR9 on indigenous bacteria in the cucumber rhizosphere, we grew cucumber seedlings with and without SQR9 in a pot experiment using sterilized (SS) and natural (NS) soils (Figure 1A). Inoculation with *B. velezensis* SQR9 significantly stimulated cucumber seedlings growth in both soils compared with that of the non‐inoculated control seedlings (Figure S1). Furthermore, cucumber plants growth was stronger in NS than in SS (Figure S1).

**FIGURE 2:**
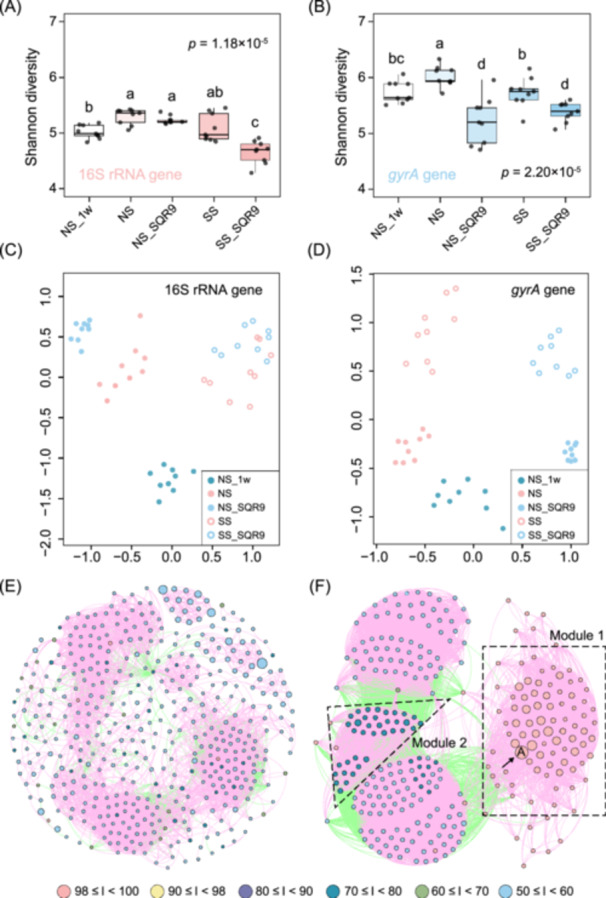
Alteration of the bacterial community composition in the cucumber rhizosphere by *B. velezensis* SQR9 via high‐throughput 16S rRNA gene and *gyrA* sequencing. (A) Shannon diversity indices of the 16S rRNA gene sequence‐based bacterial communities. (B) Shannon diversity indices of the *gyrA* gene sequence‐based bacterial communities; different letters above the boxes indicate significant differences between experimental variants, as determined by the Kruskal–Wallis test followed by the Mann–Whitney *U* test. (C) Nonmetric multidimensional scaling plot of taxonomic similarity of the 16S rRNA gene (Bray–Curtis). (D) Nonmetric multidimensional scaling plot of the compositional similarity of the *gyrA* gene (Bray–Curtis). (E) *gyrA* gene co‐occurrence network of the non‐SQR9‐treated rhizosphere *Bacillus* and its related bacterial communities (non‐sterilized soil [NS] and sterilized soils [SS]). (F) *gyrA* gene co‐occurrence network of the SQR9‐treated rhizosphere *Bacillus* and its related bacterial communities (SS_SQR9 and NS_SQR9). Nodes with different colors depict the level of relatedness between *B. velezensis* SQR9 *gyrA* gene and other *gyrA* gene sequences. Capital A indicates the node of *B. velezensis* SQR9 in the community. The letter I represents the nucleotide identity of the *gyrA* gene. Modules 1 and 2 show the two groups of nodes clustered together in the community after inoculation with SQR9. For E and F, pink lines indicate positive correlations and green lines indicate negative correlations. *gyrA*, DNA gyrase subunit A gene.

To obtain detailed information on how strain SQR9 influenced the resident bacterial community in the rhizosphere, we analyzed the diversity of the bacterial community using 16S rRNA gene amplicon sequencing (Figure 1B). We further analyzed the *gyrA* gene abundance of *Bacillus* species, which showed increased phylogenetic resolution for the diversity of *Bacillus* community [46]. Using both 16S rRNA gene and *gyrA* gene analyses, we showed that the addition of SQR9 to SS (SS_SQR9) significantly reduced the Shannon diversity index (H‐index) of total bacterial communities in the rhizosphere (Figure 2A) as well as that of *Bacillus* and its related bacterial communities (Figure 2B). In contrast, in NS soils inoculated with SQR9 (NS_SQR9), no reduction in the H‐index was observed for total bacterial communities (Figure 2A); however, a reduction was detected in *Bacillus* and its related bacterial communities (Figure 2B). In the nonmetric multidimensional scaling (NMDS) analysis, the amplicon data of both the 16S rRNA and *gyrA* genes showed that inoculation of strain SQR9 into the rhizosphere significantly affected the bacterial community in cucumber rhizosphere. Compared with that of the 16S rRNA gene (statistical ANOSIM *R* = 0.370, *p* < 0.001; Figure 2C), the *gyrA* NMDS analysis showed a more distinct separation between the rhizosphere soil samples treated with SQR9 and those untreated (statistical ANOSIM *R* = 0.424, *p* < 0.001; Figure 2D). In the 16S rRNA‐based compositional analysis, treatment with SQR9 significantly altered the rhizosphere microbial community. At the phylum/class level, the relative abundance of Firmicutes markedly increased by 3788.0% and 48.1%, while that of Gammaproteobacteria increased by 88.2% and 56.5% under the NS_SQR9 and SS_SQR9 treatments, respectively (Figure S2A). At the family level, Bacillaceae abundance showed a substantial increase of 71.8% under NS_SQR9 and 64.2% under SS_SQR9. Burkholderiaceae abundance increased by 127.2% in NS and by 23.7% in SS, whereas Cytophagaceae abundance decreased by 34.8% in NS and by 58.7% in SS (Figure S2B). At the genus level, *Bacillus* was notably enriched, with its relative abundance increasing by 7550.0% under NS_SQR9 and by 50.6% under SS_SQR9 treatment (Figure S2C).

Next, we conducted a network analysis of the *gyrA* gene co‐occurrence patterns to investigate the interactions between SQR9 and the indigenous rhizosphere *Bacillus* and its related bacteria community, as well as their impact on community assembly. In the SQR9‐treated rhizosphere, we observed a reduction in the total number of nodes and an increase in the number of positive and negative links (Spearman's correlation coefficient *ρ* > 0.80, *p* < 0.01, two‐sided tests; Table S1). Additionally, SQR9 only accounted for 4% of the *gyrA* reads, and there was sufficient read depth both with and without SQR9 to construct a co‐occurrence diagram (Figure S3A–C). SQR9 addition strongly affected the co‐occurrence network of the indigenous rhizosphere *Bacillus* and its related bacterial communities (Table S1 and Figure 2E,F). Specifically, the degree of relatedness between the SQR9‐*gyrA* and other *gyrA* sequences confirmed a shift in community structure and enrichment of specific bacterial taxa in the rhizosphere (Figure 2E,F). For example, in the untreated soil network, blue nodes representing phylogenetically distant members (50% ≤ identity [I] < 60%) dominated (Figure 2E). In contrast, we observed enrichment of both highly phylogenetically related (Module 1, 98% ≤ I < 100%) and moderately phylogenetically related members (Module 2, 70% ≤ I < 80%) with a concomitant increase in node connections in the SQR9‐treated soil (Figure 2F). Moreover, the number of distantly related members decreased in the SQR9‐inoculated samples (Figure 2F and Table S2). Similar patterns were observed regardless of whether the analysis was performed with or without SQR9 reads (Figure S3D,E). Overall, these results indicated that SQR9 altered the composition of the indigenous bacterial community in the rhizosphere, with most significant shifts occurring in the genus *Bacillus* and its relatives compared to those in other bacteria. This suggested that SQR9 may specially have interacted with and shaped the *Bacillus* and its related bacterial communities, promoting the enrichment of more closely related members within the rhizosphere *Bacillus* community.

### *B. velezensis* SQR9 inoculation enhances the compatibility and cooperative behavior of *Bacillus* communities in the rhizosphere

To examine whether SQR9 increases the compatibility of the *Bacillus* rhizosphere community, we isolated 180 spore‐forming *Bacillus* strains and the related genera (hereafter referred to as “*Bacillus* strain”) from SS and SS_SQR9 cucumber rhizospheres, through three rounds of selection, with 30 strains selected from each round for every treatment (Figure 1C). Compatibility was tested using a swarm encounter assay with 2610 pairwise strain combinations (excluding self‐pairs). Isolates were selected based on their swarming ability and biofilm formation, both of which are essential for host‐plant interactions. A boundary at the swarm encounter indicated antagonism, and merging swarms suggested compatibility [35, 39].

**FIGURE 3:**
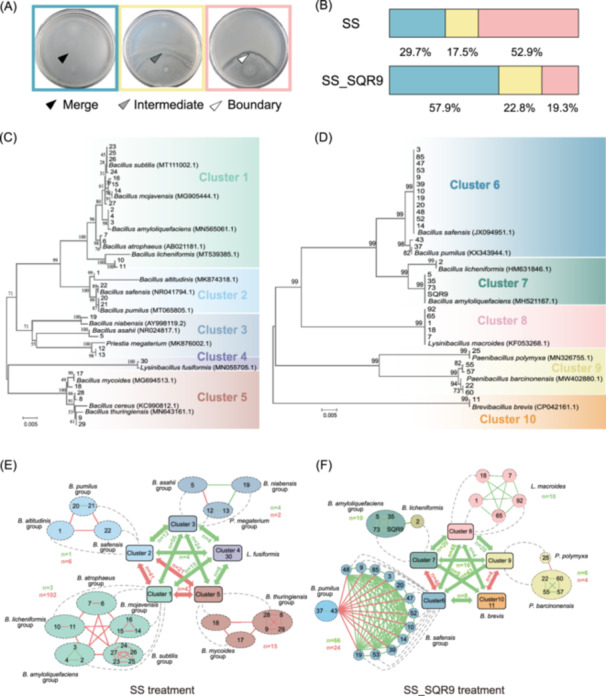
Soil inoculation with the strain *B. velezensis* SQR9 modifies the distribution of pairwise swarm interaction phenotype patterns of isolates representing *Bacillus* community in the rhizosphere. (A) Examples of merging or boundary formation between *Bacillus* strains isolated from cucumber rhizosphere and inoculated to swarm toward each other on a 9‐cm‐wide plate. Merging phenotype (black arrow); intermediate boundary (gray arrow), strong boundary (white arrow). Intermediate phenotype (gray mark) indicates a less striking but still visible line at the swarm meeting point. (B) Ratios of swarm interaction phenotypes (merging, intermediate, and boundary) among 30 *Bacillus* isolates from SS and SS_SQR9 treatments (see Table S3 for detail). (C) Minimum‐evolution tree based on *gyrA* gene sequences from 30 strains isolated from cucumber rhizosphere in the SS treatment. (D) Minimum‐evolution tree based on *gyrA* gene sequences from the 30 strains isolated from the cucumber rhizospheres of the SS_SQR9 treatment. The tree was constructed using MEGA (v.5.05) for Minimum‐evolution, and the reliability of clades was tested using the 1000 bootstrap replications. Swarm interaction network of the strains isolated from cucumber rhizospheres of the (E) SS treatment and (F) SS_SQR9 treatment. The dashed lines indicate the taxonomic clusters associated with each ecological group. Green connection lines represent strains with merging phenotype; red connection lines represent boundary formation. Colors depict different groups, *n* represents the number of pairwise combinations displaying swarm interaction phenotypes within group or between clusters (numbers in green color indicate the number of merging phenotypes, and numbers in red color indicate the number of boundary phenotypes).

We then compared the frequency of swarming phenotypes (merging, intermediate, or boundary; Figure 3A) between strains from SS and SS_SQR9 rhizosphere soils, using 60 strains isolated in each round of selection, with the pairing performed for three rounds (replicates). The first data set showed that SQR9 inoculation increased the frequency of the swarm‐merging phenotype in *Bacillus* sp. isolates. In the SS rhizosphere, only 29.7% of pairwise combinations merged, whereas 58.9% of pairs from SQR9‐treated rhizospheres merged their swarms. Additionally, the frequency of boundary formation was lower in the SQR9‐treated soils (19.3%) than in SS (52.9%) (Figure 3B and Table S3). The other two datasets corroborated the findings of the first set, i.e., the frequency of boundary formation was consistently lower in SQR9‐treated soils (17.5% in replicate 2 and 22.1% in replicate 3) than in the untreated soils (55.2% in replicate 2 and 57.5% in replicate 3) (Figure S4). These findings were consistent with the bioinformatics data (Figure 2E,F), suggesting that SQR9 altered the *Bacillus* and its related bacterial communities toward a more compatible and potentially cooperative behavior.

To link the different swarm patterns observed in the SS and SS_SQR9 rhizospheres with the phylogenetic relatedness of interacting strains, we first determined the pairwise nucleotide identities of the *gyrA* gene among the strains. We then constructed phylogenetic trees for the strains isolated from the SS (Figure 3C and Table S4) and SS_SQR9 treatments (Figure 3D and Table S5). These isolated strains are collectively referred to as the isolated communities. Compared with that of the SS_SQR9‐treatmented samples, the diversity of bacteria screened from the SS‐treated samples was high; however, the isolates from the SS‐treated samples displayed the swarm merging phenotype less frequently (Figure 3E,F). Merging was the predominant phenotype in the strains from SS_SQR9‐treated samples, with *gyrA* gene nucleotide identities ranging from 96% to 99.5% (Figure 3D,F).

For strains under the SS treatment, the boundary phenotype was predominant within the arbitrary clusters (boundaries [*B*] = 125, merging [*M*] = 8), with merging and boundary formation between clusters being nearly equal (*B* = 105, *M* = 121; Figure 3E). Among the strains isolated from SS_SQR9 plants, the merging phenotype dominated among species clusters and between arbitrary clusters (*B* = 28, *M* = 92). Although certain strains of two closely related species within arbitrary clusters formed boundaries (e.g., *Paenibacillus polymyxa* and *P. barcinonensis* or *B. safensis* and *B. pumilus*), some also merged (e.g., *B. licheniformis* and *B. amyloliquefaciens*). Moreover, we observed an increase in merging among strains from different arbitrary clusters (*B* = 52, *M* = 164; Figure 3F). Overall, these observations suggested that SQR9 rhizosphere inoculation reduced the frequency of antagonistic species and increased the compatibility of the rhizosphere bacterial community.

### Construction of MR consortia to promote plant growth

The above‐mentioned results showed that *B. velezensis* SQR9 altered the composition and social interactions of the rhizosphere *Bacillus* and its related bacterial communities, thereby enriching both HR and MR strains that exhibited swarming compatibility (Figure 2E,F). These findings provide valuable insights for further construction of our synthetic PGP consortia. Resource competition among member strains is a key consideration in PGP consortia design and is often influenced by strain relatedness. Understanding how to combine these two factors to optimize PGP consortia is an important research question that requires further investigation.

**FIGURE 4:**
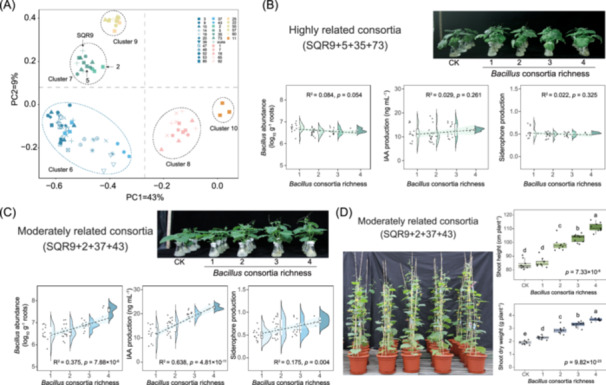
Carbon resource competition and sociality are two important parameters for smart design of PGP *Bacillus* consortia. (A) Principal component analysis (PCA) of carbon source utilization pattern on Biolog GEN III plates of 30 strains isolated from cucumber rhizospheres (SS_SQR9 treatment). The plotted data are averages of three independent experiments. The location of *Bacillus* isolates used to build consortia is marked. Briefly, *B. velezensis* 5, 35, 73, and SQR9 were used to build HR consortia; and *B. licheniformis* (2), *B. pumilus* (37 and 43) and *B. velezensis* (SQR9) were used to build MR consortia. (B) Construction of HR consortia and the richness effect on functions. The consortia were constructed using strains SQR9, 5, 35, and 73. Varying richness (1–4) of the consortia was used to explore the effects of levels on cucumber root colonization, IAA production, and siderophore production. (C) Construction of MR consortia and the richness effect on functions. The consortia were constructed using strains SQR9, 2, 37, and 43. Varying richness (1–4) of the consortia was used to explore the effects of levels on cucumber root colonization, IAA production, and siderophore production. CK is the treatment without inoculation of strains. Statistical analysis was performed using generalized linear models (GLM) in R (v.4.2.0). *p* Values for the regression coefficients were calculated to evaluate the relationships between *Bacillus* consortia richness and PGP properties across different analyses. *R*² values were used to assess the goodness of fit of the GLM. (D) Effects of MR consortia with varying richness levels on cucumber growth in a natural soil system. Different letters above the boxes indicate significant differences. Statistical analyses were performed using the Kruskal–Wallis test followed by the Mann–Whitney *U* test for shoot height, and Tukey's honestly significant difference (HSD) test following one‐way analysis of variance (ANOVA) for shoot dry weight.

To test our hypothesis, we first investigated resource competition among the candidate PGPR strains in our consortia. The carbon source utilization of 30 strains from the rhizosphere treated with SQR9 was measured using the GEN III MicroPlate test assay performed using a Biolog system. Closely related strains exhibited greater similarity in utilizing carbon sources than the compatible but MR strains (Figure S5). Moreover, principal component analysis (PCA) showed that the patterns of carbon source utilization strongly correlated with the phylogenetic relatedness of *Bacillus* isolates (Figure 4A).

To verify the effect of strain relatedness on the PGP activity of consortia, we compared two types of consortia with four richness levels—the HR swarm‐compatible consortia (100% nucleotide identities of the *gyrA* gene, composed of isolates 5, 35, 73, and SQR9) and MR swarm‐compatible consortia (70%–80% nucleotide identities of the *gyrA* gene, composed of isolates 2, 37, 43, and SQR9; Figures 1D, 3D, 4A, and S6, Table S6). The consortium design was conceived to ensure that each isolate was present at comparable frequencies at each diversity level, allowing for the separation of the effects of bacterial richness and composition [12]. The selection criteria were as follows: for HR consortia, we selected strains with a merging phenotype and high carbon resource competitiveness; for MR consortia, we selected strains with a merging phenotype and low carbon resource competitiveness. The PGP potential of individual isolates was assessed, predicting that at a richness level of 4, the PGP potential of the two *Bacillus* consortia with related phylogeny was comparable (Figure S7). Increasing the richness of the PGP consortia is known to positively affect PGP activity in tomato plants [12]. Thus, we tested the effect of increasing richness for MR and HR consortia on cucumber growth both in an experimental hydroponic system and in potting experiments with natural soil. We did not observe any increase in shoot height and dry weight in HR consortia with increasing richness compared to that in the control (CK) experiments in a sterile system (fold change [FC, richness 4/CK] = 0.97, *p* = 0.191; FC = 0.99, *p* = 0.361). Additionally, PGP activities in consortia, including root colonization and IAA and siderophore production (FC [richness 4/richness 1] = 0.98, *R* ^2^ = 0.084, *p* = 0.054; FC = 1.12, *R* ^2^ = 0.029, *p* = 0.261; FC = 0.97, *R* ^2^ = 0.022, *p* = 0.325), did not show any significant changes with increasing richness, regardless of whether one or multiple strain consortia were used (Figures 4B and S8A,B). Consistent with our predictions, the MR consortia led to a significant increase in the shoot heights and shoot dry weights of cucumbers (FC [richness 4/CK] = 1.51, *p* < 0.001; FC = 1.79, *p* < 0.001; Figure S8C,D). Furthermore, root colonization and IAA production in MR consortia were also increased with increasing richness levels (FC [richness 4/richness 1] = 1.16, *R*² = 0.375, *p* < 0.0001; FC = 1.93, *R*² = 0.638, *p* < 0.0001; Figure 4C). The effect of MR consortia richness on siderophore production was also significant (FC [richness 4/richness 1] = 1.63, *R*² = 0.175, *p* < 0.01; Figure 4C). Additionally, the MR consortia showed enhanced plant growth and yield not only in hydroponic culture systems but also in potting experiments conducted using unsterilized natural soil substrates (Figure 4D). In conclusion, these results indicated that mixing MR swarm‐merging strains significantly promoted PGP activity in a richness‐dependent manner, highlighting the importance of relatedness‐dependent ecological compatibility and niche breadth when designing consortia.

### Validation of consortia design strategy through enhanced richness and combinatorial variations

The *Bacillus* consortium design strategy, which considers the phylogenetic relatedness of member strains within the consortium, was found to enhance the overall PGP functions of the consortium. However, due to the limited number of consortium combinations tested, the general applicability of this strategy remained unconfirmed. Therefore, we expanded the experimental design by increasing the richness and number of consortium combinations to further validate the strategy. We built *Bacillus* consortia using a substitutive design with increased richness levels of 1, 2, 4, and 8 strains from a collection of 60 *Bacillus* isolates. We designed 150 combinations with each consortium type, 60 with a single strain, 30 with two strains, 30 with four strains, and 30 with eight strains (Figures 1E, 3C,D and Table S7). The consortium design principles in this section were similar to those in the previous section. The rhizosphere colonization ability of the consortia was assessed in a hydroponic system, and their IAA and siderophore production capacities were measured using cocultivation methods to evaluate their PGP activity.

**FIGURE 5:**
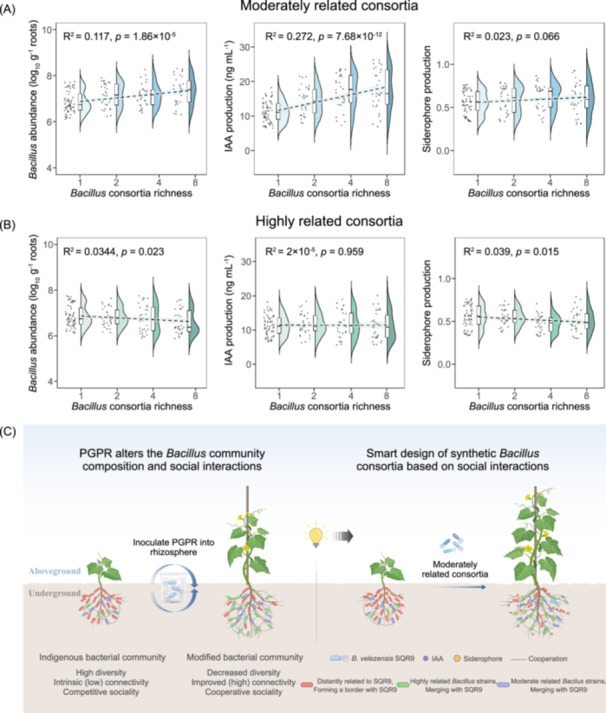
Validation of the consortium construction strategy and the proposed synthetic *Bacillus* consortia design based on social interactions. (A) Effects of increasing richness (from 1 to 8 strains) in MR consortia on cucumber root colonization, IAA production, and siderophore production were evaluated across 150 combinations. (B) Effects of increasing richness (from 1 to 8 strains) in HR consortia on cucumber root colonization, IAA production, and siderophore production were assessed using 150 consortium combinations. Statistical analysis was performed using generalized linear models (GLM) in R (v.4.2.0); *p*‐values for the regression coefficients represent the relationships between *Bacillus* consortia richness and PGP properties across different analyses; *R*² values reflect the goodness of fit of the GLM. (C) Schematics for the impact of plant‐growth promoting rhizobacteria (SQR9) on the *Bacillus* and its related bacteria community and the design of synthetic *Bacillus* consortia based on social interactions. Left: Strain SQR9 alters the *Bacillus* and its related bacteria community toward more cooperative and compatible behavior in cucumber rhizospheres, leading to the promotion of plant growth. Right: Smart design of synthetic *Bacillus* consortia based on the social interactions observed in the modified community. The plant growth‐promoting ability of MR consortia was stronger than that of HR consortia. IAA, indole‐3‐acetic acid; PGPR, plant growth‐promoting rhizobacteria.

We hypothesized that the HR and MR consortia would differently influence colonization abundance, IAA and siderophore production, and overall PGP properties. Consistent with the trends observed in the first batch of consortia (Figure 4), the MR consortia demonstrated enhanced PGP activity, including increased *Bacillus* abundance (FC [richness 8/richness 1] = 1.07, *R* ^2^ = 0.117, *p* < 0.0001) and IAA production (FC = 1.56, *R* ^2^ = 0.272, *p* < 0.0001), in the hydroponic experimental system, with these improvements becoming more evident as richness increased (Figure 5A). The high variability in results prevented us from detecting significant correlations in experiments with eight different strains tested in 150 combinations; however, the trend of increasing PGP activity with increasing richness was validated (Figure 5B). These richness‐dependent effects were not observed for the HR consortia, as no significant changes were found in *Bacillus* abundance (FC [richness 8/richness 1] = 0.96, *R* ^2^ = 0.0344, *p* = 0.023), IAA production (FC = 0.99, *R* ^2^ = 0.00002, *p* = 0.959), or siderophore production (FC = 0.92, *R* ^2^ = 0.039, *p* = 0.015). We concluded that the positive effect of MR consortium richness on PGP activity may have been due to the low competition among individuals and the potential synergies created by complementary niches leading to more effective use of available resources (Figure 5A,B).

## DISCUSSION

Plant‐associated beneficial microorganisms show considerable promise for improving crop quality and productivity [7, 47, 48]. However, their use has been hampered by our limited understanding of the effects of relationships among microbial inoculants on the plant and its rhizosphere microbiome [49]. In this study, *B. velezensis* SQR9 shifted the composition of indigenous rhizosphere bacteria, particularly *Bacillus* species, toward a less competitive and more cooperative community, resulting in the enrichment of HR and MR strains (Figure 5C). To the best of our knowledge, these results provide the first evidence that associated sociality shifts community‐level functionality, as reflected in the improved PGP activities of MR consortia. This finding is crucial for developing more reliable PGP inoculants.

### SQR9 application alters the composition of the *Bacillus* community and related species in the rhizosphere

*Bacillus* species are known for their PGP activities and are widely used in agroecosystems to improve plant health [50, 51]. However, the impact of these agents on the resident rhizosphere bacterial community has remained debated. Previous studies examining the effects of inoculants, such as *B. velezensis* FZB42, *B. velezensis* BNM122, and *B. subtilis* PTS‐394, using amplicon sequencing of 16S rRNA genes did not observe a significant impact on bacterial community composition [52, 53, 54]. However, in this study, we complemented 16S rRNA gene analysis with *gyrA* gene amplicon sequencing. Although we observed a decrease in 16S rRNA gene diversity in the SS_SQR9 rhizosphere, the *gyrA*‐targeting approach revealed previously undetected remarkable changes in the composition of *Bacillus* species and closely related genera (e.g., *Paenibacillus*, *Brevibacillus*, and *Lysinibacillus*), emphasizing the importance of intra‐ and interspecific competition. This approach required the development of a new tool, the *gyrA*3 primer pair, which targets the diversity of *Bacillus* species and its closely related strains [46]. Our study demonstrated that this primer successfully amplified the *gyrA* gene from the rhizosphere bacterial community associated with an agriculturally important plant. Moreover, by targeting lower taxonomic units, we showed that amplicon sequencing enhanced the detection and resolution of rhizosphere microbiota dynamics. Additionally, this study demonstrated that SQR9 induced a significant shift toward a less diverse community, with a larger proportion of highly interconnected strains that increased in frequency. These strains were either HR (98%–100% identities, red group) or MR (70%–80% identities, green group), as shown in Figure 2F. In contrast, genotypes with 50%–60% *gyrA* identities (blue group) decreased in frequency. SQR9 also increased the number of connections within each group, revealing a positive correlation among the close relationship nodes (Figure 2E,F).

### Strain SQR9 changes social interactions in the rhizosphere bacterial community

Although previous research has highlighted the importance of rhizosphere microbiome for plant health [1, 55] and the potential benefits of bioinoculants are widely recognized [5, 7], the effects of bioinoculants on bacterial social interactions in the rhizosphere have remained largely ignored. This study showed that strain SQR9 induced a dramatic shift in the community, increasing the proportion of closely related and interconnected strains (Figure 2E,F). Furthermore, overall compatibility among isolates from the rhizosphere treated with SQR9 increased dramatically (Figure 3B). The patterns of the *gyrA* gene co‐occurrence and the results of swarming assays provided the first evidence that *Bacillus* and its closely related species were important targets of SQR9 activity in the rhizosphere (Figures 2E,F and S6, Table S2).

What mechanisms might underlie these effects? Niche reshaping based on secondary metabolites is one potential mechanism. For example, the untreated isolated rhizosphere community included 15 *Bacillus* species and only one *Lysinibacillus* isolate (Figure 3C). In contrast, the SQR9‐treated community consisted of four *Bacillus* species and three MR species (Figure 3D), confirming the strong antagonism of SQR9 against closely related species. *B. velezensis* strains (including SQR9) produce a variety of bioactive secondary metabolites [56, 57, 58, 59, 60], which may lead to intense competition and niche emptying and consequently provide opportunities for compatible strains to thrive. This line of reasoning is consistent with our results.

KD explains the enrichment of compatible strains (Figure 3A,B) [17]. In *Bacillus* species, KD involves intercellular attack and defence molecules with varying gene combinations across strains [38]. Antagonistic actions are evident from the appearance of visible boundaries between swarming strains [35]. Strains that form boundaries on semisolid media typically are unable to coexist on plant roots, while swarming mergers join to form a plant root biofilm. Killing competitors can provide an advantage; indeed, we observed that strain SQR9 promoted the enrichment of compatible strains within specific clusters (Figure S6) and reduced diversity within species clusters (Figure 3C,D). Although swarm merging is generally associated with *Bacillus* intraspecific sociality (including all intraspecies references) and some more distantly related *Bacillus* species [38], we found that MR genera in the rhizosphere (including *Bacillus*, *Paenibacillus*, and *Lysinibacillus*) also merged their swarms.

By examining the community from two perspectives (i.e., how the addition of strain SQR9 affects community relatedness and compatibility with SQR9, and how the strains in the rhizosphere interact with each other after SQR9 inoculation; Figures 2 and 3), we concluded that strain SQR9 promoted cooperativity within the community. However, it remains unclear whether this shift toward enhanced cooperation in the community was transient or long‐lasting. Overall, our results demonstrated that SQR9, through antagonism, created an opportunity for compatible strains to thrive and consequently reduce competition. These findings are consistent with the currently established theory that a certain level of competition is essential for the development of cooperation in bacteria [27, 61].

Moreover, although our work is the first to investigate the impact of rhizosphere inoculation of PGPR strains on social interactions of rhizosphere bacteria, social interactions of rhizosphere microorganisms may be more complex than we initially anticipated. For example, quorum sensing [62], secretion of secondary metabolites [63, 64], and contact‐dependent inhibition effects based on various secretion systems [65, 66] can all influence social interactions. These aspects need to be gradually expanded upon the basis of KD.

### Smart design of synthetic *Bacillus* consortia with defined ecological (social) interactions

Plant‐associated microbial communities have numerous potential applications in biotechnology, particularly in agriculture [67]. Recently, microbial consortia with lower complexity have been studied and used as model systems for controlled assessment of ecological, structural, and functional properties of microbial communities [68]. However, rational engineering of beneficial consortia with robust survivability and activity for reliable field applications is a major challenge [69]. Addressing this challenge requires integrating multiple approaches, including reverse engineering of natural communities (e.g., inference‐based co‐occurrence analysis) and rational design of consortia with desirable interactions that enhance community‐level functionality and robustness [70]. While several underlying factors could directly or indirectly affect the performance of synthetic *Bacillus* consortia in the rhizosphere [71], our results showed that sociality and competition for carbon among these strains are critical parameters to consider when developing efficient plant probiotics (PGP inoculants). Our results also showed that mixing compatible strains that did not compete for the same resources resulted in more efficient PGP inoculants. This was confirmed by their PGP activity in both hydroponic and natural soil systems (Figure 4C,D). A trade‐off between cooperation and competition within synthetic consortia may also exist. While HR consortia may exhibit strong cooperative PGP traits driven by KD, intense resource competition may constrain their long‐term coexistence. In contrast, MR consortia may strike a more favorable balance between cooperation and competition, potentially facilitating a more stable and resilient community structure.

Although this study examined the social interactions of rhizosphere bacteria in a natural soil system, we did not assess the broader effects of SQR9 inoculation on the entire microbial community. The plant rhizosphere microbiota encompasses all living members of the microbiome, including bacteria, archaea, fungi, protists, and algae [72]. However, the focus of the present study was to elucidate the social relationships among bacteria, particularly those from the genus *Bacillus* and related genera. Therefore, a key challenge for future research is to address the social interactions among different types of microorganisms in the rhizosphere, including fungi and archaea, which may provide more comprehensive insights into the effects of microbial inoculants [73].

## CONCLUSION

Overall, this study provides a general ecological framework for intelligent assembly of *Bacillus* consortia for more efficient and reliable applications. When designing microbial synthetic community products, it is essential to consider social interactions among microorganisms, particularly with regard to *Bacillus* species. In this study, we first showed that the application of the beneficial strain SQR9 shifted the bacterial community (particularly the *Bacillus* and its related bacteria community) toward increased cooperativity. By studying how strain SQR9 altered the indigenous microbial patterns in the cucumber rhizosphere, we designed MR *Bacillus* consortia and confirmed their PGP effects in both sterile hydroponic systems and natural soil pot cultures. Finally, our results suggested that incorporating the ecological mechanisms (sociality and competition for carbon) used by microbial inoculants can aid in improving guidelines and lead to the development of more effective products for sustainable agriculture.

## MATERIALS AND METHODS

### Plant cultivation, DNA extraction, sequencing, and data analysis

Two‐week‐old cucumber seedlings from a hydroponic culture system were transferred to pots filled with 200 g of natural soil and incubated for 1 week, and then DNA from rhizosphere soil was extracted from 9 out of 45 treatments (NS_1w) (Figure 1A). The remaining seedlings were transferred to pots filled with 200 g of natural (NS) or sterilized soil (SS). Importantly, cucumber seedlings were not vigorously shaken before transferring the plants to allow the roots to carry the rhizosphere soil, which ensured that a large amount of rhizosphere microbial community was transferred into both soil types (SS and NS). Then, both soil types were inoculated with 10 mL suspensions (10^7^ CFU mL^−1^) of *B. velezensis* SQR9 (NS_SQR9 and SS_SQR9) and incubated for an additional 2 weeks; at the same time controls without the SQR9 inoculation were treated the same way. At this point, DNA was extracted from the rhizosphere soil for 16S rRNA and *gyrA* gene amplicon sequencing (Figure 1B). Rhizosphere soil from SS and SS_SQR9 was also used for *Bacillus* spp. strain isolation (Figure 1C).

Cucumber rhizosphere soils were collected as described by Chaparro et al. [74]. Total DNA was extracted from 0.25 g of rhizosphere soil using PowerSoil DNA Isolation Kit (Mo Bio Laboratories). A NanoDrop ND‐2000 spectrophotometer (Thermo Scientific) was used to assess DNA quality [75]. Amplification of the V3–V4 hypervariable region of the bacterial 16S rRNA gene was performed to assess the bacterial community using the primers 338F: 5′‐CCTACGGRRBGCASCAGKVRVGAAT‐3′ and 806R: 5′‐GGACTACNVGGGTWTCTAATCC‐3′. For *Bacillus* and its close relatives, amplification of the *gyrA* gene was performed using the primers 243F: 5′‐GCDGCHGCNATGCGTTAYACTC‐3′ and 736R: 5′‐CGGACAAGMTCWGCKATTTTTTC‐3′ to assess the community composition [46]. Nine 16S rRNA gene samples and three *gyrA* gene samples were sequenced per treatment using an Illumina MiSeq instrument (Illumina, Inc.).

Sequencing profiles of the 16S rRNA and *gyrA* genes were processed using the UPARSE pipeline (http://drive5.com/usearch/manual/uparse_pipeline.html) [76]. Paired‐end sequences were merged utilizing “fastq_mergepairs,” followed by high‐quality sequence selection via “fastq_filter.” After singleton and chimeric sequences being removed, amplicon sequence variants (ASVs) in 16S rRNA gene profiles were generated using the Greengenes 16S rRNA gene database (released in May 2013, https://ftp.microbio.me/greengenes_release/gg_13_8_otus/). The ASV profiles in *gyrA* genes in this study were not annotated against any database. Following the exclusion of ASVs classified as “Chloroplasts,” “Mitochondria,” and “Archaea,” the ASV table was normalized using “otutab_norm” to 5000 reads per sample, resulting in 321,536 high‐quality reads for the 16S rRNA gene sequencing and 236,687 high‐quality reads for the *gyrA* gene sequencing.

The relative abundance of all *gyrA* genes was used for network analysis, which was performed using the Molecular Ecological Network Analyses Pipeline (MENAP) (http://ieg4.rccc.ou.edu/mena/main.cgi) [77]. The analysis involved two steps: network construction and analysis. Network construction included data updates, standardization, pairwise similarity of relative abundance across samples, and determination of the adjacency matrix using an RMT‐based approach. Network analysis encompassed module detection, overall topological structure, and topological role identification of individual nodes [78]. Shannon diversity index calculation and Bray–Curtis dissimilarity‐based NMDS analysis were conducted on the rarefied sequencing data using the vegan R package (v.2.5‐2) (https://cran.r-project.org/package=vegan).

### Strain isolation and screening

Rhizosphere soils from the SS and SS_SQR9 treatments (Figure 1A) were resuspended in 1 mL of sterile saline solution (0.9% NaCl) and heated at 80°C for 15 min to kill vegetative cells while preserving spores. The resulting spore suspensions were plated on tryptose blood agar and incubated at 30°C for 24 h. Emergent colonies were streaked three times to obtain pure cultures, yielding 280 isolates. From these, 30 representative isolates from each of the SS and SS_SQR9 treatments were selected based on the following criteria: (a) three metabolic tests (catalase test, Voges‒Proskauer test, and anaerobic growth on agar) and 16S rRNA gene nucleotide identity identified them as *Bacillus* [79]; (b) the ability to form pellicles (floating biofilm) in MSgg medium [80]; (c) the ability to swarm on B‐medium with 0.7% agar (glucose 2 g L^−^¹, (NH_4_)_2_SO_4_ 1.982 g L^−^¹, MgSO_4_ 0.963 g L^−^¹, KCl 2.013 g L^−^¹, sodium citrate 1.807 g L^−^¹, Tris·HCl 7.880 g L^−^¹, CaCl_2_ 0.222 g L^−^¹, FeSO_4_ 0.000152 g L^−^¹, MnSO_4_ 0.00151 g L^−^¹, KH_2_PO_4_ 0.0817 g L^−^¹, sodium glutamate 0.761 g L^−^¹, lysine 0.126 g L^−^¹, tryptophan 0.159 g L^−^¹, pH 7.5) [35]. The top 30 isolates, ranked by their swarming ability, were selected from both SS and SS_SQR9 treatments.

### Swarm boundary assay

To test the social interaction between approaching swarms of different *Bacillus* spp., isolates were inoculated onto B‐medium plates with 0.7% agar and cultured in 3 mL of liquid B medium and shaken overnight at 30°C. These cultures were then diluted to an optical density (OD_600_) of 0.5, and 2 μL was spotted on the plates at each side of the agar plate. The plates were dried in a laminar flow hood for 20 min, incubated for 2 d at 30°C, and photographed. Three phenotypes (merging, intermediate, and boundary) were assigned to 870 pairs of swarms as described previously [35].

### Phenotypic characterization

Ammonia (NH_3_) production was assessed using 1.5% peptone water, where isolates were grown at 30°C with shaking for 2 d, then supplemented with 5% Nessler reagent. The resulting color change was measured at a wavelength of 425 nm, and NH₃ production was quantified using a standard curve of pure NH₃ (0–100 mM) [81]. Growth was assessed in 200 μL of minimal MGY medium (glucose 5 g L^−^¹, yeast extract 4 g L^−^¹, NH₄NO₃ 1 g L^−^¹, NaCl 0.5 g L^−^¹, K₂HPO₄ 1.5 g L^−^¹, KH₂PO₄ 0.5 g L^−^¹, MgSO₄ 0.2 g L^−^¹, pH 7.0) in 96‐well plates, with an initial OD_600_ of 0.05, and OD_600_ readings were taken every 30 min at 30°C using a Bioscreen C system (Bioscreen C pro). Siderophore production was assayed by growing isolates in MKB medium at 30°C with shaking for 48 h, followed by mixing 0.5 mL of cell‐free supernatant with 0.5 mL chrome azurol S assay solution, with OD_630_ measured after 2 h [82, 83]. IAA production was measured after culturing isolates in liquid Landy medium (glucose 20 g L^−^¹, l‐glutamic acid 5 g L^−^¹, KH₂PO₄ 1 g L^−^¹, yeast extract 1 g L^−^¹, MgSO₄ 7H₂O 0.5 g L^−^¹, KCl 0.5 g L^−^¹, MnSO₄ H₂O 5 mg L^−^¹, CuSO₄ 7H₂O 0.16 mg L^−^¹, FeSO₄ 7H₂O 0.15 mg L^−^¹, l‐phenylalanine 2 mg L^−^¹, l‐tryptophan 1 g L^−^¹, pH 7.0) for 72 h at 22°C with shaking, followed by centrifugation at 10,000 × *g* for 2 min and IAA quantification using an ELISA kit (MEIMIAN, China) [43, 84]. Phosphate solubilization was evaluated by culturing isolates in NBRIP medium (glucose 10 g L^−^¹, Ca₃(PO₄)₂ 5 g L^−^¹, MgCl₂ 6H₂O 5 g L^−^¹, MgSO₄ 7H₂O 0.25 g L^−^¹, KCl 0.2 g L^−^¹, (NH₄)₂SO₄ 0.1 g L^−^¹, pH 7.0) for 7 days at 30°C with shaking, followed by centrifugation and measurement of soluble phosphate concentration using the molybdenum‐antimony method [85]. Each assay was repeated three times.

### Carbon source utilization assay

The carbon source utilization patterns of 30 isolates from cucumber rhizosphere soils were assessed using a GEN III MicroPlate with the Biolog system (Biolog). Bacterial suspensions were inoculated into the MicroPlate following the manufacturer's instructions (http://www.biolog.com), and carbon source utilization was measured after 12 h using Protocol A of a Biolog's MicroStation™ System [28]. This experiment was conducted in triplicate. Wells displaying colouration were scored as 1, and noncoloured wells were scored as 0 for PCA. PCA was conducted using the vegan R package (v.2.6‐4) [86], and scatter plots were visualized using the ggplot2 R package (v.3.4.0) [87]. Color reaction measurements from the three experimental replicates were averaged, and a heatmap was constructed using the pheatmap R package (v.1.0.12) [88].

### *Bacillus* **consortia design**

This study implemented the following two batches of consortia designs. First, *Bacillus* strains isolated from the SS_SQR9 rhizosphere were used to construct two types of consortia: HR and MR. Each consortium type included four richness levels (1, 2, 3, and 4 strains), resulting in a total of 30 consortia. The HR consortia were constructed using *B. amyloliquefaciens* (5), *B. amyloliquefaciens* (35), *B. amyloliquefaciens* (73), and *B. velezensis* (SQR9), and the MR consortia included *B. licheniformis* (2), *B. pumilus* (37), *B. pumilus* (43), and *B. velezensis* (SQR9) (Table S6). These consortia were tested for their PGP effects in greenhouse experiments (both hydroponic and soil systems) and phenotypic characterization (IAA and siderophore production).

Next, 60 *Bacillus* strains screened from the SS and SS_SQR9 rhizospheres were used to construct larger consortia for expanded validation. The consortia remained classified as HR and MR, with four richness levels (1, 2, 4, and 8 strains). Each consortium type included 150 combinations: 60 with 1 strain, 30 with 2 strains, 30 with 4 strains, and 30 with 8 strains, resulting in a total of 300 consortia. Detailed information on the components of the two *Bacillus* consortia is provided in Table S7. The consortia construction principle was the same as described above. These expanded consortia were then tested for their PGP effects in a hydroponic system and phenotypic characterization (IAA and siderophore production).

### Greenhouse experiments

Two greenhouse experiments were conducted to test the PGP effects of different *Bacillus* consortia in vivo. The first used a hydroponic culture system, in which cucumber seeds (Jinchun 4) were surface disinfected in 2% sodium hypochlorite (NaClO) for 15 min, washed with sterile water, and planted in sterile vermiculite. The seeds germinated for 4 d in a growth chamber at 23°C with a 16‐h light and 8‐h dark photoperiod. When two cotyledons appeared, the seedlings were transplanted into aseptic conical flasks containing 50 mL of ¼ sterile MS medium. Four days later, plants were inoculated with the *Bacillus* community at an OD_600_ of 0.02 and incubated on an orbital shaker at 50 rpm in a growth chamber at 28 ± 2°C (day) and 22 ± 2°C (night) for an additional 2 weeks. Each conical flask was a biological replicate, with nine replicates for each treatment and a non‐inoculated control. Plant shoot height and dry weight were measured 3 weeks later.

The second experiment was conducted in a natural soil system from August 17 to November 8, 2019, in the greenhouse at Nanjing Agricultural University. The soil was collected from a cucumber‐cultivated field site in Nanjing, Jiangsu Province, China, and had the following properties: pH 6.4, organic matter 18.6 g kg^−^¹, available N 121 mg kg^−^¹, available P 56 mg kg^−^¹, and available K 89 mg kg^−^¹. Two‐week‐old cucumber seedlings were transplanted into pots containing 5 kg of soil. After 7 d, plants were inoculated with the assembled consortia by drenching the pots to a final concentration of 10⁷ CFU g^−^¹ soil. Each treatment was replicated 18 times across three blocks, with nine seedlings randomly planted within each block. Pots were incubated in a growth chamber at 70% humidity, with natural light and temperatures of 28 ± 2°C during the day and 22 ± 2°C at night. Plants were irrigated with ½ Hoagland medium as described by Qiu et al. [89]. After 55 days, nine randomly selected plants per treatment were harvested, and shoot height and dry weight were measured.

### Root colonization assay

A cucumber hydroponic experiment was conducted. Two days after inoculation, cucumber roots with colonized cells were aseptically removed, washed, and placed on filter paper to remove planktonic cells. Roots were then shaken in a 250 mL sterile flask with 45 g of glass beads and 100 mL of sterile water for 10 min to detach the cells. Finally, the cells were counted using the dilution plate counting method [90].

### Statistical analysis

Figures were produced using the GraphPad Prism 8 or ggplot2 R package (v.4.2.0). Detailed statistical analyses are described in the figure legends. Data on all individual *Bacillus* in vitro performances (five PGP traits) were standardized between 0 (minimum value across all treatments) and 1 (maximum value across all treatments) and used in subsequent calculations and analyses as described above.

## AUTHOR CONTRIBUTIONS

**Yan Liu**: Funding acquisition; writing—original draft; writing—review and editing; visualization; software. **Baolei Jia**: Writing—review and editing. **Yi Ren**: Software; data curation. **Weibing Xun**: Software; data curation; visualization. **Polonca Stefanic**: Validation; visualization. **Tianjie Yang**: Methodology. **Youzhi Miao**: Investigation. **Nan Zhang**: Investigation; resources. **Yanlai Yao**: Resources. **Ruifu Zhang**: Funding acquisition. **Zhihui Xu**: Writing—original draft; writing—review and editing; methodology; visualization. **Qirong Shen**: Resources; funding acquisition. **Ines Mandic‐Mulec**: Writing—review and editing; visualization; resources.

## CONFLICT OF INTEREST STATEMENT

The authors declare no conflicts of interest.

## ETHICS STATEMENT

No animals or humans were involved in this study.

## Supporting information


**Figure S1.** Inoculation of *B. velezensis* SQR9 significantly stimulated cucumber growth in both natural (NS) and sterilised (SS) soils compared with their non‐inoculated controls.
**Figure S2.** Effect of *B. velezensis* SQR9 on the indigenous rhizosphere bacterial community based on 16S rRNA gene amplicon data.
**Figure S3.** Abundance of SQR9 reads across different treatment groups.
**Figure S4.** Two additional biological replicates of *Bacillus* populations isolated from SS and SS_SQR9 treatments, and the ratios of swarm interaction phenotypes (merging, intermediate, and boundary) among isolates in different treatments.
**Figure S5.** Carbon‐source utilisation of 30 strains from the rhizosphere treated with *B. velezensis* SQR9.
**Figure S6.** (A) Minimum‐evolution tree based on both 16S rRNA gene and (B) full‐length *gyrA* gene sequences from the 30 strains isolated from cucumber rhizospheres of SQR9 treatment.
**Figure S7.** Measurement of five plant growth‐promoting (PGP) traits of *Bacillus* strains used for building HR and MR consortia shown in Figures 4B and 4C.
**Figure S8.** Effect of MR consortia (SQR9+2+37+43) strain richness on cucumber growth.

**Table S1.** Topological properties of the gene co‐occurrence networks and their respective identically sized random networks.
**Table S2.** Annotation information using the NCBI database for each node in Figures 2E and 2F based on the sequencing fragment (490 bp) in gene co‐occurrence networks analysis. The similarity of the *gyrA* gene for each node to SQR9 was also present.
**Table S3.** Detailed information about swarm interaction phenotypes for *Bacillus* isolates from both SS and SS_SQR9 treatments.
**Table S4.** The *gyrA* gene sequence similarity between each pair of strains isolated from the SS treatment.
**Table S5.** The *gyrA* gene sequence similarity between each pair of strains isolated from the SS_SQR9 treatment.
**Table S6.** Detailed information of the components of 30 HR and MR consortia.
**Table S7.** Detailed information of the components of 300 HR and MR consortia.

## Data Availability

Amplicon sequencing reads from the 16S rRNA gene and *gyrA* gene are available at NCBI Sequence Read Archive under accession number, PRJNA879238 https://www.ncbi.nlm.nih.gov/bioproject/PRJNA879238/. The 16S rRNA and *gyrA* gene sequences of all isolated strains have been submitted under BioProject PRJNA1248407 https://www.ncbi.nlm.nih.gov/bioproject/PRJNA1248407/. The data and scripts used are saved in GitHub https://github.com/yanliu2023/iMeta/. Supplementary materials (figures, tables, graphical abstract, slides, videos, Chinese translated version, and update materials) may be found in the online DOI or iMeta Science http://www.imeta.science/.
