# Supplementary material for: Bacterial social interactions in synthetic Bacillus consortia enhance plant growth
Source: Imeta. 2025 Jun 8;4(4):e70053. doi: 10.1002/imt2.70053 (PMC12371272; doi:10.1002/imt2.70053)
Supplement: Supplementary file 1 — Figure S1. Inoculation of B. velezensis SQR9 significantly stimulated cucumber growth in both natural (NS) and sterilised (SS) soils compared with their non‐inoculated controls. Figure S2. Effect of B. velezensis SQR9 on the indigenous rhizosphere bacterial community based on 16S rRNA gene amplicon data. Figure S3. Abundance of SQR9 reads across different treatment groups. Figure S4. Two additional biological replicates of Bacillus populations isolated from SS and SS_SQR9 treatments, and the ratios of swarm interaction phenotypes (merging, intermediate, and boundary) among isolates in different treatments. Figure S5. Carbon‐source utilisation of 30 strains from the rhizosphere treated with B. velezensis SQR9. Figure S6. (A) Minimum‐evolution tree based on both 16S rRNA gene and (B) full‐length gyrA gene sequences from the 30 strains isolated from cucumber rhizospheres of SQR9 treatment. Figure S7. Measurement of five plant growth‐promoting (PGP) traits of Bacillus strains used for building HR and MR consortia shown in Figures 4B and 4C. Figure S8. Effect of MR consortia (SQR9+2+37+43) strain richness on cucumber growth. [file IMT2-4-e70053-s002.docx]

**Supporting information to**

**Bacterial social interactions in synthetic *Bacillus* consortia enhance plant growth**

**Running title:** *Bacillus* interactions in synthetic consortia

Yan Liu^1, 2#^, Baolei Jia^2#^, Yi Ren^1#†^, Weibing Xun^1^, Polonca Stefanic^3^, Tianjie Yang^1^, Youzhi Miao^1^, Nan Zhang^1^, Yanlai Yao^2^, Ruifu Zhang^1^, Zhihui Xu^1*^, Qirong Shen^1*^, Ines Mandic-Mulec^3*^

^1^Jiangsu Provincial Key Lab of Solid Organic Waste Utilisation, Jiangsu Collaborative Innovation Centre of Solid Organic Wastes, Educational Ministry Engineering Centre of Resource-Saving Fertilizers, Jiangsu Provincial Key Laboratory of Coastal Saline Soil Resources Utilisation and Ecological Conservation, Nanjing Agricultural University, Nanjing, 210095, China

^2^Xianghu Laboratory, Hangzhou, 311231, China

^3^Department of Microbiology, Biotechnical Faculty, University of Ljubljana, Ljubljana, 1000, Slovenia

^†^Present address: Yi Ren, Jiangsu Engineering Research Center for Soil Utilization & Sustainable Agriculture, Jiangsu Centre for Collaborative Innovation in Geographical Information Resource Development and Application, School of Geography, Nanjing Normal University, Nanjing, 210023, China

^#^These authors contributed equally: Yan Liu, Baolei Jia, and Yi Ren

*Correspondence: [xzh2068@njau.edu.cn](mailto:xzh2068@njau.edu.cn) (Zhihui Xu), shenqirong@njau.edu.cn (Qirong Shen), and [Ines.MandicMulec@bf.uni-lj.si](mailto:Ines.MandicMulec@bf.uni-lj.si) (Ines Mandic-Mulec)


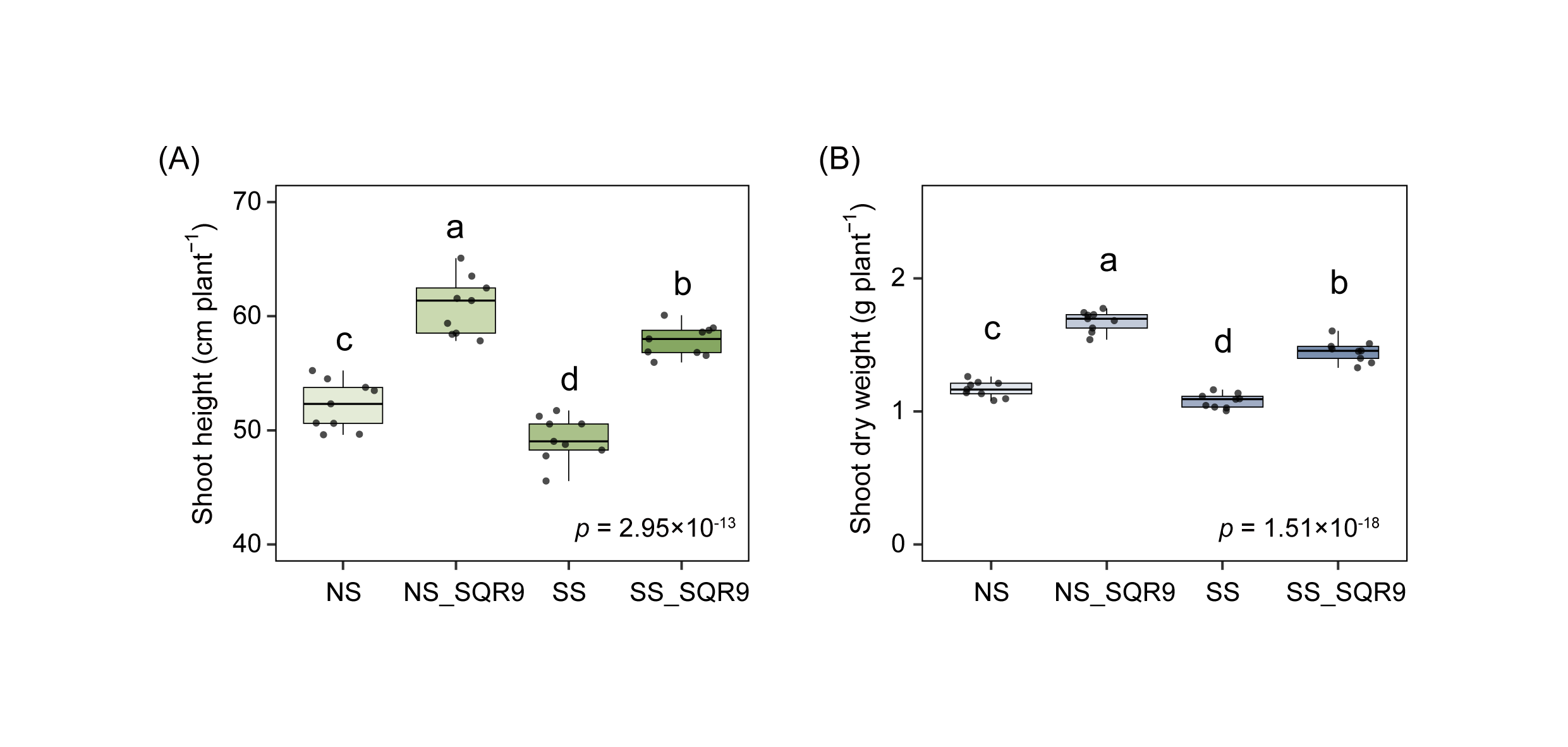


**Figure S1** Inoculation of *B. velezensis* SQR9 significantly stimulated cucumber growth in both natural (NS) and sterilised (SS) soils compared with their non-inoculated controls. (A) Inoculation of SQR9 significantly increases cucumber shoot height. (B) Inoculation of SQR9 significantly increases cucumber shoot dry weight. Different letters above the boxes indicate significant differences between experimental variants, as determined by the one-way ANOVA followed by the Tukey’s Honestly Significant Difference test.

**
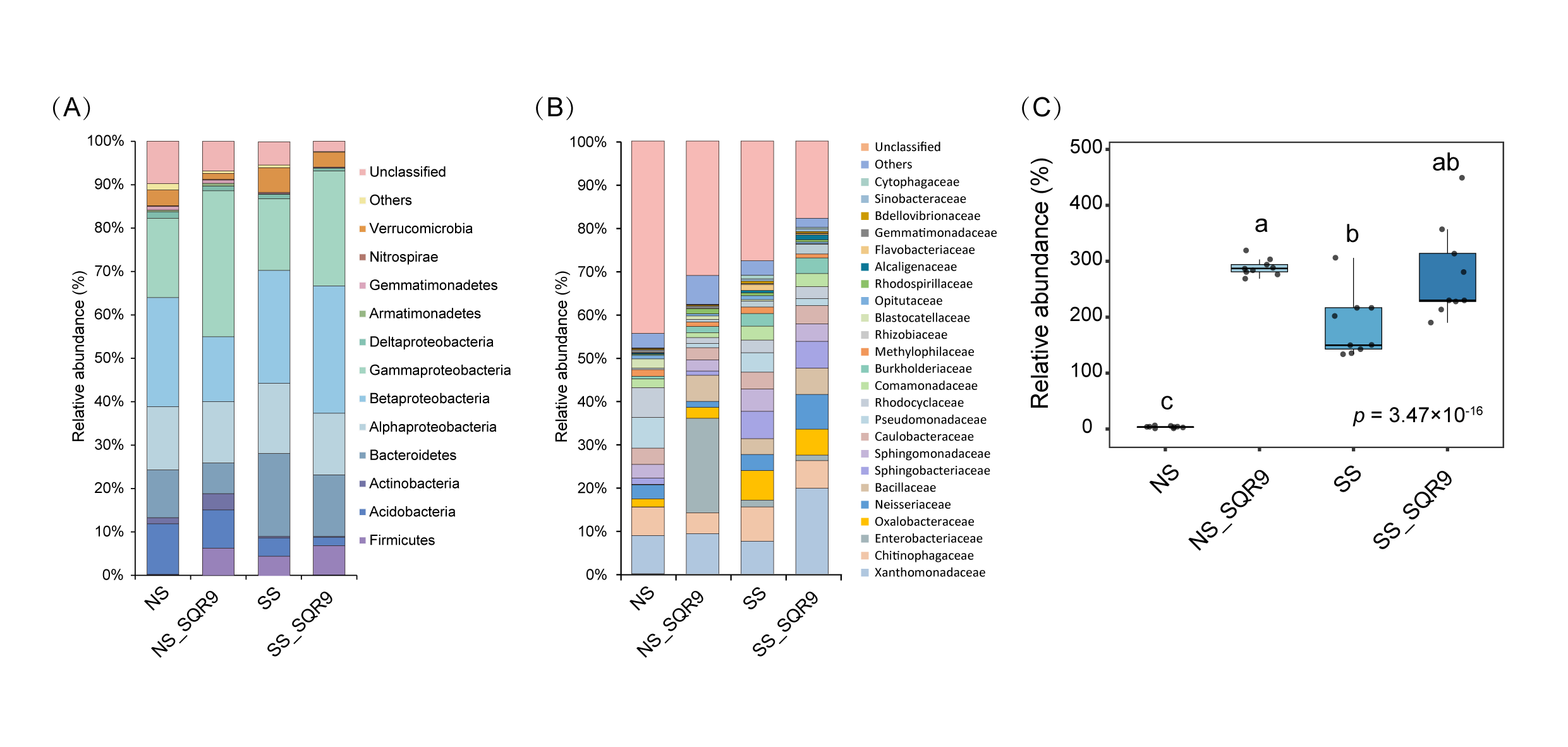
**

**Figure S2** Effect of *B. velezensis* SQR9 on the indigenous rhizosphere bacterial community based on 16S rRNA gene amplicon data. (A) Distribution of 16S rRNA gene sequences across bacterial phylum/class in different treatments. (B) Distribution of 16S rRNA gene sequences across bacterial families in different treatments. (C) Relative abundance of the *Bacillu*s genus in different treatments. Different letters above the boxes indicate significant differences between experimental variants, as determined by the Welch’s ANOVA followed by the Games–Howell test.


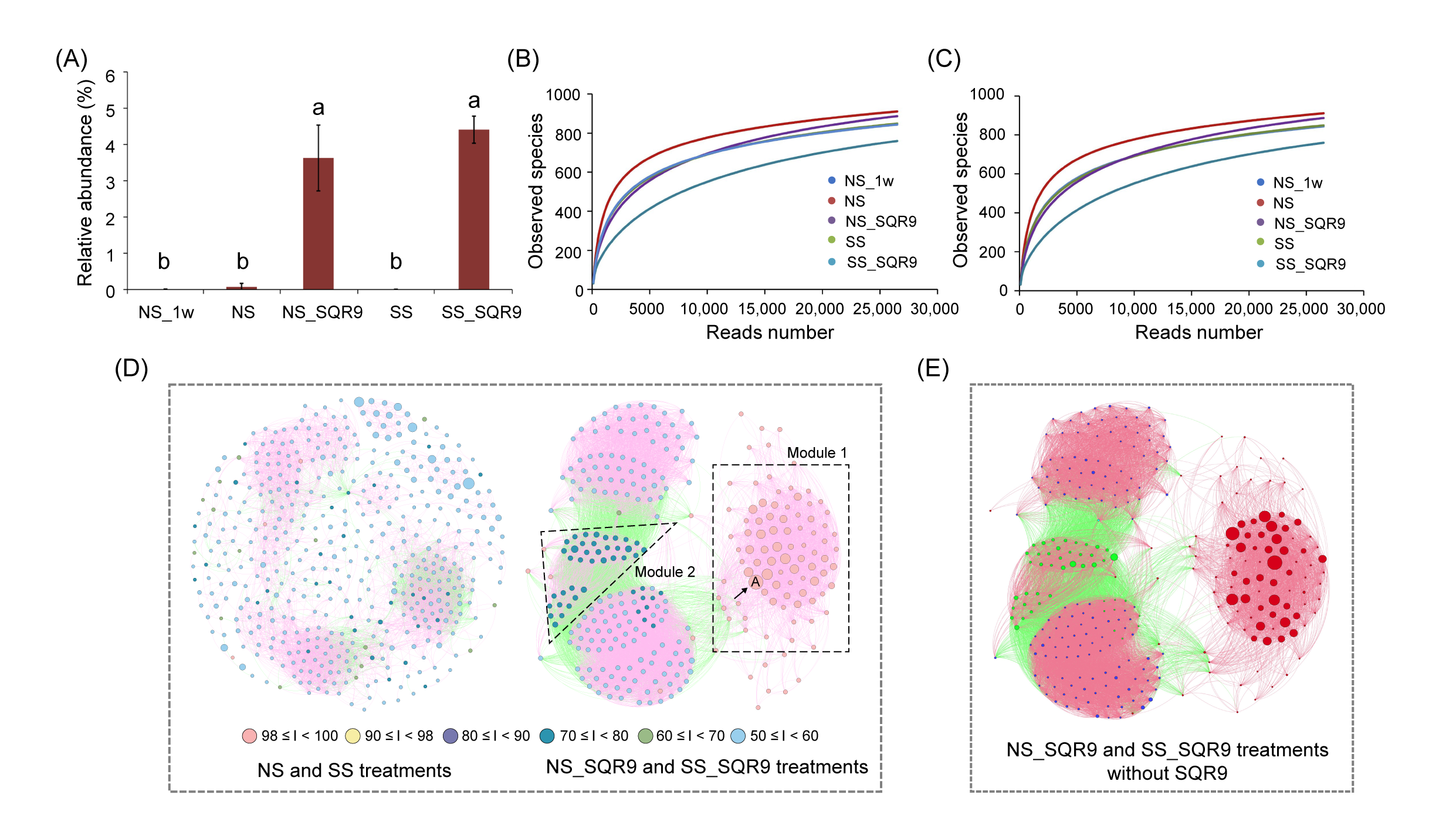


**Figure S3** Abundance of SQR9 reads across different treatment groups. (A) Bioinformatics analyses with or without SQR9 reads based on *gyrA* gene amplicon data. Different letters above the bars indicate significant differences between experimental variants, as determined by the one-way ANOVA followed by the Tukey’s HSD test (*p <* 0.05, *n* = 9, mean ± standard deviation). Rarefaction curves of the bacterial *gyrA* gene data sets with (B) and without (C) SQR9 reads. The lines for NS_1w and SS almost overlap each other. The patterns of co-occurrence networks are similar whether SQR9 reads are included (D) or not (E).


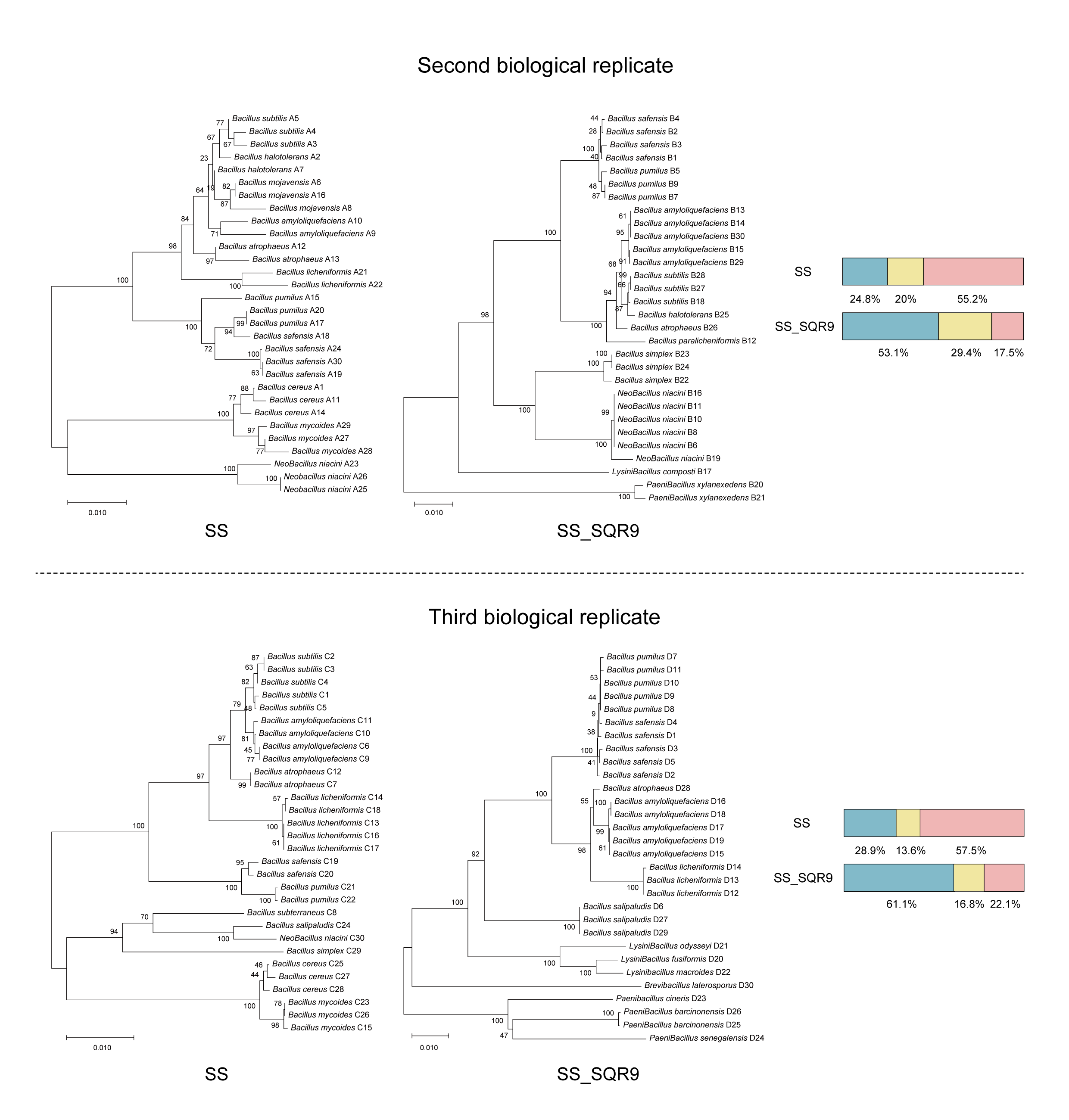


**Figure S4** Two additional biological replicates of *Bacillus* populations isolated from SS and SS_SQR9 treatments, and the ratios of swarm interaction phenotypes (merging, intermediate, and boundary) among isolates in different treatments.


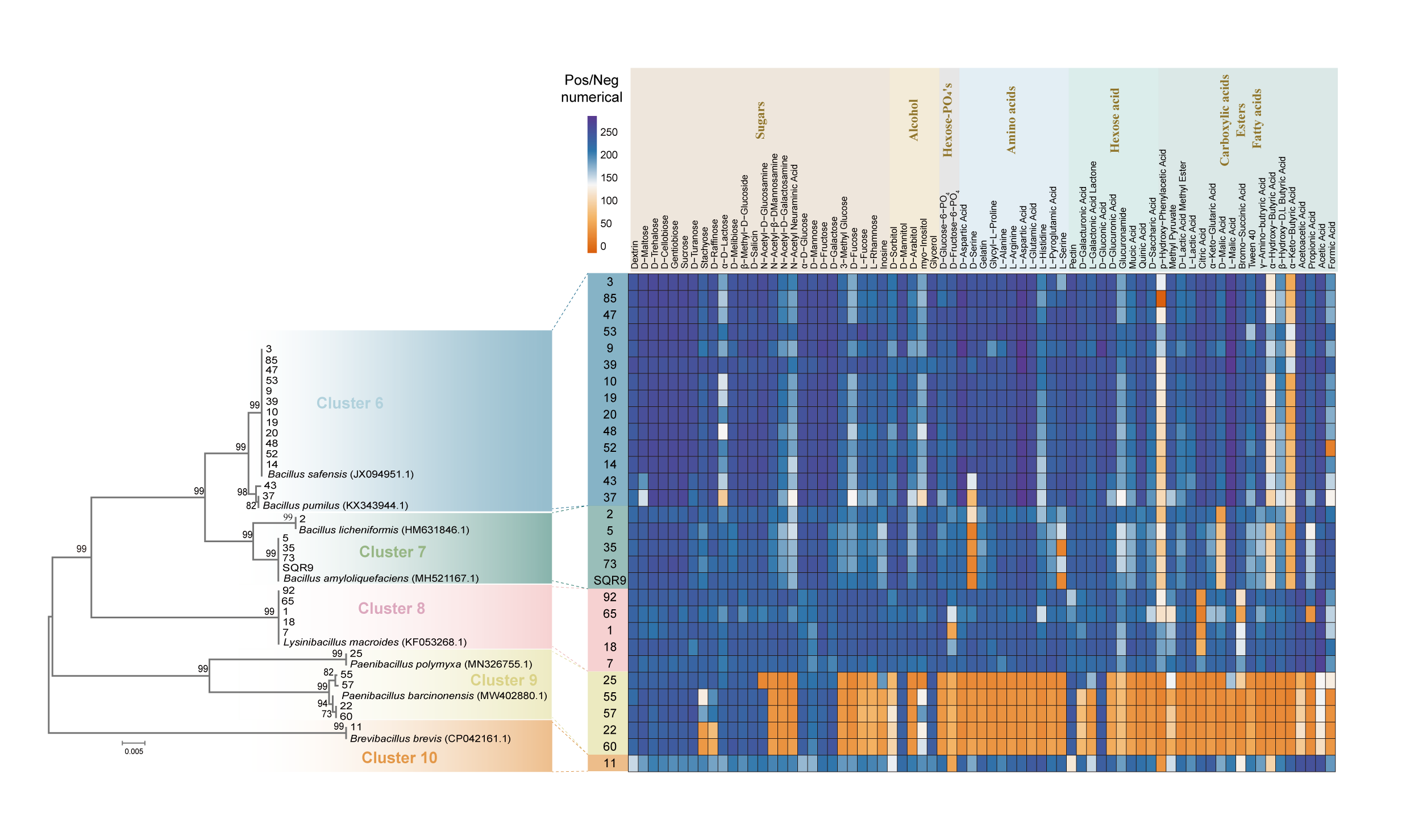


**Figure S5** Carbon-source utilisation of 30 strains from the rhizosphere treated with *B. velezensis* SQR9.


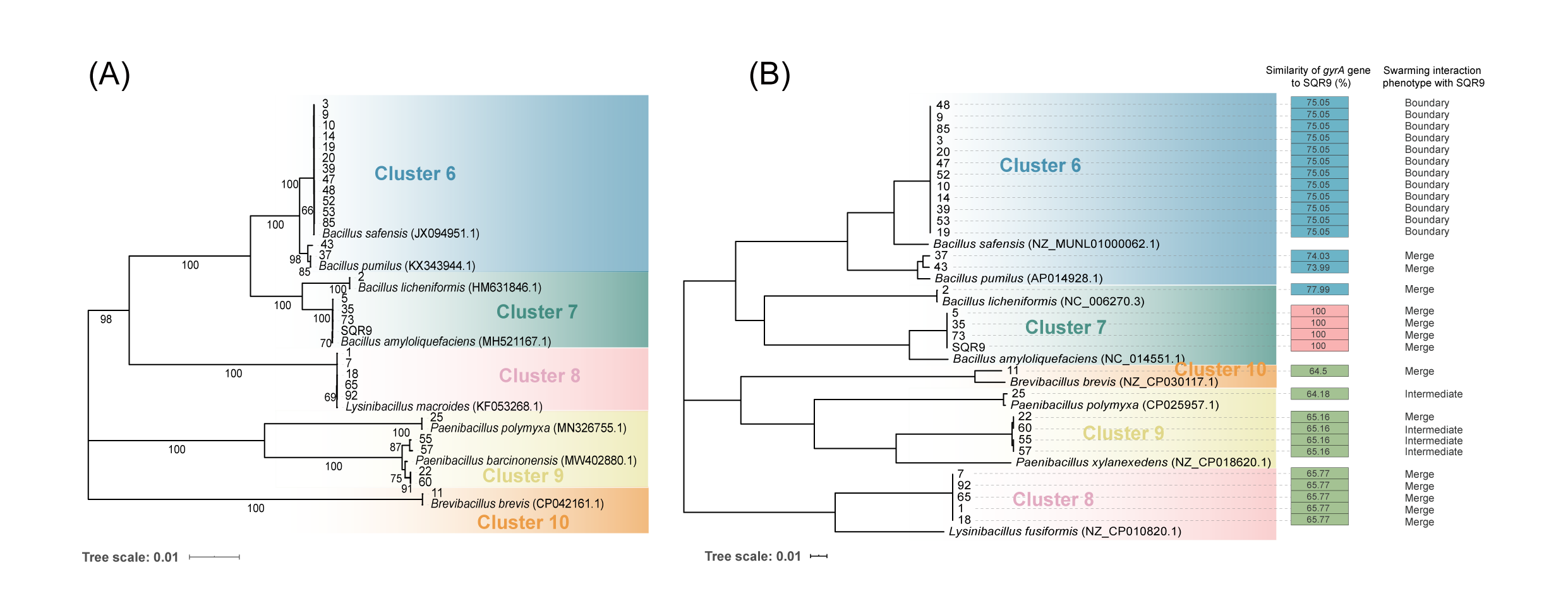


**Figure S6** (A) Minimum-evolution tree based on both 16S rRNA gene and (B) full-length *gyrA* gene sequences from the 30 strains isolated from cucumber rhizospheres of SQR9 treatment. For each isolate, the similarity of *gyrA* gene to SQR9 and the swarm interaction phenotype with SQR9 are also indicated in the figure.


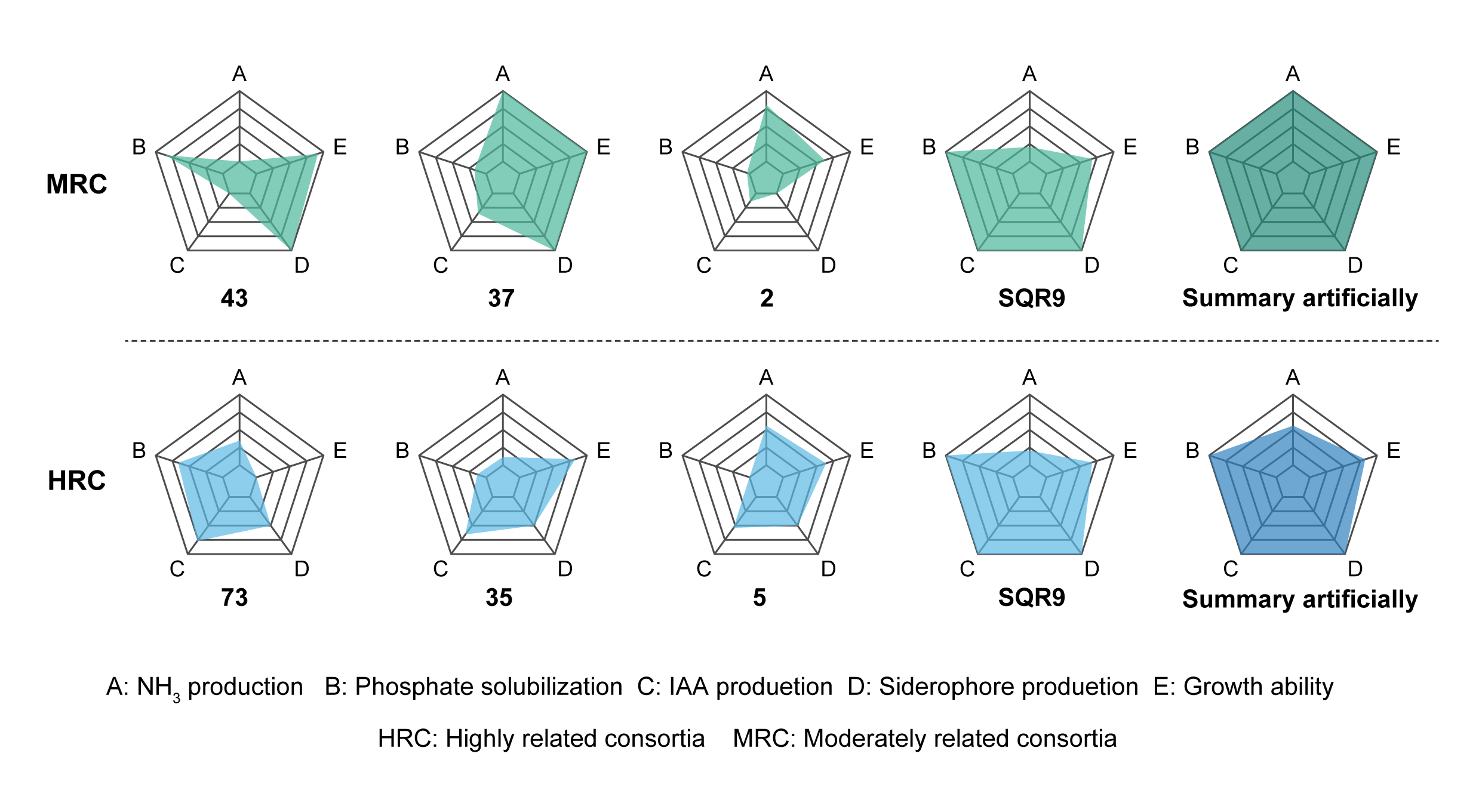


**Figure S7** Measurement of five plant growth-promoting (PGP) traits of *Bacillus* strains used for building HR and MR consortia shown in Figure 4B and 4C. Each measurement was assigned a relative value between 0 and 1 based on the min-max measured values for each trait. The last “summary artificially” pentagons indicate the total PGP trait prediction for the *Bacillus* consortium as a whole. IAA, indole-3-acetic acid.


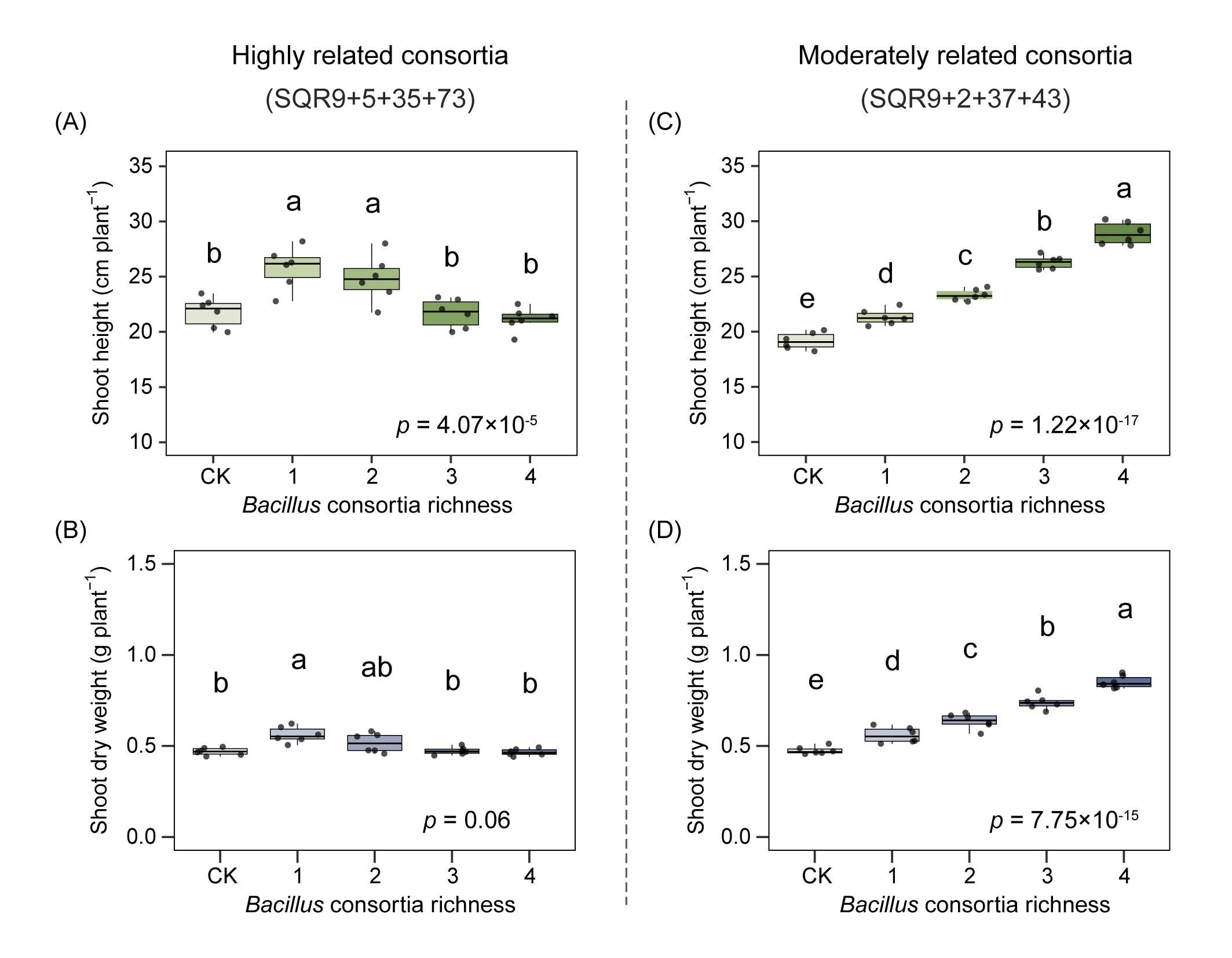


**Figure S8** Effect of MR consortia (SQR9+2+37+43) strain richness on cucumber growth. Increasing richness of HR consortia (SQR9+5+35+73) had no effect on cucumber shoot height (A) and shoot dry weight (B) in the sterile hydroponic culture system. In contrast, increasing richness of MR consortia (SQR9+2+37+43) added stepwise improvements to the cucumber shoot height (C) and shoot dry weight (D) under the same conditions. Different letters above the boxes indicate significant differences between experimental variants, as determined by the Welch’s ANOVA followed by the Games–Howell test in panel (B), and by the one-way ANOVA followed by the Tukey’s HSD test in panels (A), (C), and (D).
